# *Candida albicans* Factor H Binding Molecule Hgt1p – A Low Glucose-Induced Transmembrane Protein Is Trafficked to the Cell Wall and Impairs Phagocytosis and Killing by Human Neutrophils

**DOI:** 10.3389/fmicb.2018.03319

**Published:** 2019-01-15

**Authors:** Samyr Kenno, Cornelia Speth, Günter Rambach, Ulrike Binder, Sneha Chatterjee, Rita Caramalho, Hubertus Haas, Cornelia Lass-Flörl, Jutamas Shaughnessy, Sanjay Ram, Neil A. R. Gow, Dorothea Orth-Höller, Reinhard Würzner

**Affiliations:** ^1^Division of Hygiene and Medical Microbiology, Medical University of Innsbruck, Innsbruck, Austria; ^2^Division of Molecular Biology, Medical University of Innsbruck, Innsbruck, Austria; ^3^Division of Infectious Diseases and Immunology, University of Massachusetts Medical School, Worcester, MA, United States; ^4^School of Biosciences, University of Exeter, Exeter, United Kingdom

**Keywords:** fungal infections, *Candida albicans*, complement system, immune evasion, factor H

## Abstract

Complement is a tightly controlled arm of the innate immune system, facilitating phagocytosis and killing of invading pathogens. Factor H (FH) is the main fluid-phase inhibitor of the alternative pathway. Many pathogens can hijack FH from the host and protect themselves from complement-dependent killing. *Candida albicans* is a clinically important opportunistic yeast, expressing different FH binding molecules on its cell surface, which allow complement evasion. One such FH binding molecule is the transmembrane protein “High affinity glucose transporter 1” (Hgt1p), involved in glucose metabolism. This study demonstrated that Hgt1p transcription and expression is induced and highest at the low, but physiological glucose concentration of 0.1%. Thus, this concentration was used throughout the study. We also demonstrated the transport of Hgt1p to the fungal cell wall surface by vesicle trafficking and its release by exosomes containing Hgt1p integrated in the vesicular membrane. We corroborated Hgt1p as FH binding molecule. A polyclonal anti-Hgt1p antibody was created which interfered with the binding of FH, present in normal human serum to the fungal cell wall. A chimeric molecule consisting of FH domains 6 and 7 fused to human IgG1 Fc (FH6.7/Fc) even more comprehensively blocked FH binding, likely because FH6.7/Fc diverted FH away from fungal FH ligands other than Hgt1p. Reduced FH binding to the yeast was associated with a concomitant increase in C3b/iC3b deposition and resulted in significantly increased *in vitro* phagocytosis and killing by human neutrophils. In conclusion, Hgt1p also exhibits non-canonical functions such as binding FH after its export to the cell wall. Blocking Hgt1p-FH interactions may represent a tool to enhance complement activation on the fungal surface to promote phagocytosis and killing of *C. albicans.*

## Introduction

*Candida albicans* is a normal resident of the human oral cavity, the gastrointestinal tract (Strijbis et al., 2014) and vaginal mucous membranes (Peters et al., 2014). The human immune system plays a key role in inhibiting the proliferation of *C. albicans* in colonized areas. However, in an immunocompromised host, *C. albicans* can lead to local or systemic opportunistic infections (Musial et al., 1988). Virulence factors such as surface-expressed or secreted proteins play a pivotal role in enabling *C. albicans* to facilitate host blood dissemination (Mayer et al., 2013). Dimorphism is a crucial virulence factor of *C. albicans* that enables the fungus to change its shape in response to the external stimuli, thus facilitating its ability to colonize its host and cause invasive disease. The filamentous form empowers the fungus to induce tissue invasion and ultimately reach the blood vessels. The reversion to the yeast form enables *C. albicans* to disseminate through the bloodstream and cause multi-organ infection (Brand, 2012). The therapeutic options for invasive fungal infections have become increasingly limited (Roemer and Krysan, 2014).

Innate immunity plays a crucial role in antifungal defense. A critical arm of innate defenses is complement, a tightly controlled system which is able to recognize and kill the pathogens that breach host epithelial and mucosal barriers (Merle et al., 2015). Three different complement activation pathways commence rapidly following infection. The purpose of all three pathways is to activate C3. The resulting fragments of C3 that are generated play different crucial roles to combat invasion by microorganisms. Opsonization of the invading microbe with iC3b is critical for its phagocytosis, the most effective mechanism to clear fungi (Speth et al., 2008). The complement system has to be regulated to prevent uncontrolled activation and the attack of host cells. In the fluid phase, this regulation is mainly facilitated by complement factor H (FH), a protein composed of 20 short consensus repeats (Kopp et al., 2012). The major opportunistic fungi such as *Candida* spp. have developed a strategy to limit complement action by hijacking FH from the host by employing specific FH binding molecules on their cellular surfaces (Meri et al., 2004; Kraiczy and Wurzner, 2006; Vogl et al., 2008), thereby mimicking the host cells. Hgt1p, a transmembrane protein involved in glucose metabolism in *C. albicans* is also a FH binding molecule (Lesiak-Markowicz et al., 2011). Recently, HGTs were identified as a large family of glucose transporters in *C. albicans* (Hgt1p to Hgt20p). They share 10–93% of sequence identity and the expression of the HGTs genes is related to the glucose concentration in the growth medium (Fan et al., 2002).

Here we show that the expression of *HGT1* was highest at low (relative to enriched media), but physiological glucose concentrations, was associated with intracellular and extracellular vesicles, and also plays a non-canonical role by down-modulating phagocytosis and killing by human neutrophils. Blocking FH binding to fungi enhanced phagocytosis.

## Materials and Methods

### Reagents and Media

Fluorescein isothiocyanate (FITC), Ficoll Paque-1077, RPMI 1640 medium with L-glutamine, glucose free, HEPES without sodium bicarbonate, Phosphate Buffered Saline (PBS), TRIzole-reagent, Sodium Chloride (NaCl), Ethylenediaminetetraacetic acid (EDTA), Dithiothreitol (DTT), Tris hydrochloride (Tris-HCl), Potassium chloride (KCl), Carbonate-bicarbonate, Tween-20, D-Sorbitol, 1,4-Piperazinediethanesulfonic acid sodium salt (PIPES), protease inhibitor cocktail, Phenylmethylsulfonylfluorid (PMSF) were obtained from Sigma (St. Louis, MO, United States). Bovine Serum Albumin (BSA), peptone from soy and D-(+) glucose were purchased from Carl Roth GmbH (Karlsruhe, Germany). Yeast extract and granulated agar were obtained from BD Biosciences (San Diego, CA, United States). EvaGreen was purchased from BioRad (Hercules, CA, United States). The qScript 1-step fast Kit Reverse Transcriptase was obtained from Quanta Bioscience (Beverly, MA, United States). Goat anti-human complement FH was purchased from Calbiochem. Phycoerythrin (PE)-conjugated anti-C3b/iC3b was obtained from Biolegend (San Diego, CA, United States). Rabbit anti-goat IgG FITC conjugated was purchased from Life Technologies (Carlsbad, CA, United States). The immune coat gold goat anti-rabbit IgG 20 nm was obtained from Innova bioscience, Expedeon (San Diego, CA, United States). Zymolyase was purchased from AMS Biotechnology (Abingdon, United Kingdom). Pioloform coated copper grids were obtained from Agar Scientific (Stansted, United Kingdom).

#### Fungal Strains

The strains used in this study were *C. albicans* parental strain SN152 (an Arg^-^/Leu^-^/His^-^ auxotrophic strain), used as parental strain for knocking out *HGT1* according to standard protocols (Noble and Johnson, 2005), as well as *hgt1*Δ/Δ homozygous null mutant and *hgt1*Δ/Δ*::HGT1* complemented control strain, both generated previously (Lesiak-Markowicz et al., 2011). The strains were grown on YPD agar (Yeast extract 1%, Peptone 2%, and Dextrose 2%). Prior to all experiments, a colony each of all the strains was cultured in liquid medium YP 0.1% of glucose (Yeast extract 1%, Peptone 2%) or, in selected experiments, in YP 0.3% of glucose or YPD (containing 2% glucose) overnight (ON) at 30°C under agitation. Both, parental and mutant strains produce similar biomasses after incubation at low (0.1%) glucose concentrations. Approximately three times higher biomasses of parental and mutant strains were produced at high (2%) glucose concentrations. No differences in biomass between the two strains were detected when grown for 48 h in media containing 0.1, 0.3, or 2% of glucose (data not shown).

### Anti-Hgt1p Antibody

**FIGURE 1:**
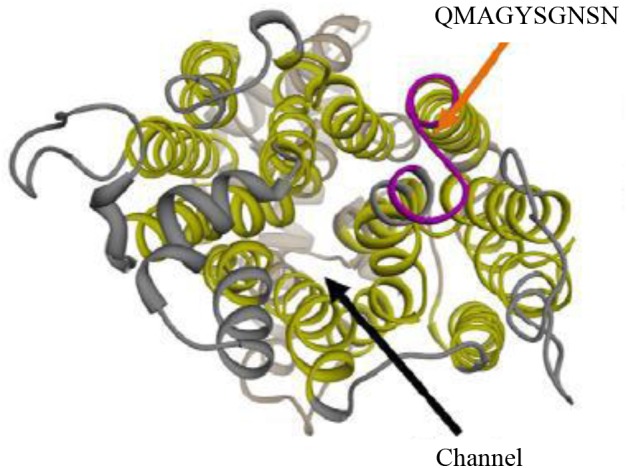
3D schematic representation of Hgt1p. The polyclonal antibody anti-Hgt1p, used in this project, was commercially generated by ProteoGenix, Schiltigheim, France. It recognizes an epitope exposed in an extracellular portion of *Candida albicans* cell membrane (upper arrows).

A polyclonal anti-Hgt1p antibody was commercially generated by ProteoGenix, Schiltigheim, France. The sequence of Hgt1p (NCBI reference XP_712952.1) was aligned with known protein structures data bank. The structure of 4GBY.PDB was selected as 29% homologous to Hgt1p. Selected structure was aligned with XP_712952.1 to identify regions that can be remodeled by sequence homology, then analyzed by 3D model to confirm the sequence on the extracellular side of *C. albicans* membrane (Figure 1). Within the sequence of Hgt1p used to generate the antibody, VMMYYIVYIFF**QMAGYSGNSN**LVASS, the peptide in bold was selected from an extracellular portion of the molecule between amino acids 306 and 315 from a total length of 545 amino acids. This synthetic peptide (location shown in Figure 1, orange and blue arrows) was used by ProteoGenix to immunize rabbits. The antibodies were used in Western blot analysis of cell membranes, cell wall extracts and whole cells of *C. albicans* SN152 in comparison with the *hgt1*Δ/Δ null mutant to validate that the antibody binds to an epitope on the former, and thus likely to Hgt1p (hence termed anti-Hgt1p), but only to a negligible extent to the null mutant (data not shown). Rabbit anti-human IgG (DAKO, Glostrup, Denmark) was used as irrelevant (rabbit) antibody and thus as a control for blocking by rabbit anti-Hgt1p. No differences in growth between the parental strain SN152 and SN152 incubated with different dilutions of anti-Hgt1p were detected over a period of 48 h (data not shown).

### FH6.7/Fc Chimeric Molecule

Cloning, expression in CHO cells and purification from cell culture supernatants of a chimeric protein comprising human FH domains 6 and 7 fused to human IgG1 Fc (FH6.7/Fc) has been described previously (Shaughnessy et al., 2014).

### mRNA Isolation and Quantitative Polymerase Reaction Chain (qPCR)

SN152 and *hgt1*Δ/Δ strains were grown in YP 0.1% glucose, YP 0.3% glucose and YPD liquid medium overnight at 30°C. The mRNA extraction was adapted from previous methods (Rio et al., 2010) and measured spectrophotometry at 260 nm; purity determined by the A260/A280 ratio. The qPCR assays were processed using 2 ng/μl of the extracted mRNA. *ACTIN-1* was used as a control. The sequences of the forward (F) and reverse (R) primers were as follows:

HGT1F:5′-ACTTCATCCATGGCTTTGGGT-3′;
HGT1R:5′-GCAGAACCGAAACCAACACC-3′
ACT1F:5′-TGGTGATGAAGCCCAATCCA-3′;
ACT1R:5′-TGGAGCTTCGGTCAACAAAAC-3′.

The conditions for *HGT1* and *ACT1* qPCR were: initial cycle 95°C/5 min, followed by 39 cycles of 95°C/10 s, annealing temperature 57°C/30 s followed by 72°C/30 s. The PCR products were analyzed by electrophoresis in 1% agarose gel. The purity of qPCR amplification was determined by melt curve analysis. The *HGT1* expression was evaluated as a relative normalized expression using *ACT1* as the housekeeping gene by the Bio-Rad CFX manager software.

### *HGT1* Sequencing

The DNA amplicons were purified with ExoSAP-IT (USB Corporation, Affymetrix, Santa Clara, CA, United States). The same primers described above were used to perform the sequencing PCR. The reaction was performed as follows: initial cycle at 96°C/1 min, followed by 25 cycles of 96°C/10 s, annealing temperature at 50°C/5 s followed by 60°C/4 min. The products were purified using BigDye XTerminator kit (Applied Biosystems, Carlsbad, CA, United States). Sequencing analysis was performed by ABI 3500 Genetic Analyzer (Applied Biosystems, Carlsbad, CA, United States). Subsequently, sequences were analyzed by Sequencing 5.2 analysis software (Applied Biosystems).

### Preparation of Yeast by High Pressure Freezing (HPF)

The SN152 and *hgt1*Δ/Δ strains were grown in YP 0.1% glucose at 30°C. One drop of each overnight culture (40 μl) was processed for high pressure freezing cryofixation by Leica EM PACT 2 (Leica Microsystems, Milton Keynes, United Kingdom), according to the guidelines of the Microscopy facility of the University of Aberdeen (Lopes-Bezerra et al., 2018).

### Immunogold Hgt1p Labeling

The grids were stained as previously described (Lopes-Bezerra et al., 2018). The coated grids were blocked by one drop (40 μl) of blocking buffer (BSA 1% and Tween 0.5% in PBS) for 20 min at RT. The grids were washed with 40 μl of washing buffer (BSA 0.1% in PBS) and incubated with 1 μg of anti-Hgt1p (100 μg/ml) in 40 μl washing buffer for 90 min at RT. Subsequently, the grids were washed with washing buffer and incubated with an appropriate gold conjugated secondary antibody (1:100) in blocking buffer. Afterward the grids were washed with PBS and stained with Uranyl acetate. Samples were visualized by transmission electron microscopy (TEM) JEM 1400 plus JEOL (Welwyn Garden City, United Kingdom) and captured by AMT UltraVUE camera (Woburn, MA, United States). The *hgt1*Δ/Δ null mutant and SN152 strains were stained with anti-Hgt1p, or with only the secondary gold-conjugated antibody to confirm antibody specificity.

### *C. albicans* Protoplast Formation and Hgt1p Gold Staining of Intracellular Secretory Pathways Vesicles

*Candida albicans* protoplasts were obtained as previously described (Cabezon et al., 2009). Briefly, the overnight culture of SN152 strain, grown in YP 0.1% of glucose at 30°C, was diluted to OD600 of 0.2 and grown at 30°C in YP 0.1% of glucose, 200 rpm for 4–6 h to reach the exponential phase (OD_600_ 1–1.6). The cells were washed twice with a solution of 100 mM Tris, 100 mM EDTA at pH 8.0 and incubated in the same buffer with 2-mercaptoethanol (1:200) for 30 min at 100 rpm at 30°C. Cells were then resuspended in a Sorbitol buffer (1 M sorbitol, 10 mM PIPES solution, pH 6.5) containing 100U Zymolyase and incubated at gentle shaking for 45 min at 30°C. The protoplasts formation was analyzed by light microscopy. Protoplasts were resuspended in lysis buffer (50 mM Tris-HCl, 1 mM EDTA, 150 mM NaCl, 1 mM DTT, 0.5 mM PMSF, protease inhibitor cocktail pH 7.5) for disruption.

Disruption was done by homogenization in lysis buffer on ice (Schuster et al., 2016). The extract was centrifuged using a bench top centrifuge Eppendorf 5415D at 10,000 *g* for 5 min. The supernatant was filtered in 0.2 μm filter and ultra-centrifuged using Optima MAX Ultracentrifuge (Beckman Coulter), TLA 120.1 rotor at 100,000 *g* for 1 h at 4°C. The pellet was resuspended in 100 μl of PBS. The vesicles were put to adhere on the copper pioloform coated grids overnight (ON) at 4°C. The vesicles were subsequently blocked with one drop (40 μl) of BSA 1% and Tween 0.5% in PBS for 20 min at room temperature (RT). The grids were washed with 40 μl washing buffer consisting of BSA 0.1% in PBS and incubated with 1 μg of anti-Hgt1p (100 μg/ml) in 40 μl of washing buffer for 90 min at RT. Subsequently, the grids were washed in washing buffer and incubated with an appropriate gold conjugated secondary antibody (1:100) in blocking buffer. The grids were washed with PBS and stained with Uranyl acetate. Samples were visualized by TEM.

### Hgt1p Gold Staining of Isolated Exosomes Released by *C. albicans*

*Candida albicans* SN152 strain was grown in 50 ml of YP 0.1% glucose medium at 30°C. The collected supernatant was filtered by 0.2 μm filter and ultra-centrifuged by Optima MAX Ultracentrifuge, rotor Type 70Ti at 100.000 *g* for 1 h at 4°C. The pellet was resuspended in PBS and stained as previously described for intracellular secretory pathway vesicles Hgt1p gold staining (Schuster et al., 2016). Sample was visualized by TEM. The percentage of exosomes and secretory vesicles that were positive for colloidal gold staining, and thus represented presence of Hgt1p, was measured.

### FH and C3b/iC3b Deposition on *C. albicans* Surface by Fluorescence Activated Cell Sorting (FACS)

SN152, *hgt1*Δ/Δ and *hgt1*Δ/Δ*::HGT1* reintegrated (both Lesiak-Markowicz et al., 2011) strains were grown ON in YP 0.1% glucose. 1 × 10^6^ yeasts were opsonized with 10% of fresh human serum (NHS) in PBS for 15 min at 37°C. In some experiments, the parental strain SN152 was blocked with 1 μg of anti-Hgt1p antibody, 1 μg of the chimeric molecule FH6.7/Fc or with 1 μg of an irrelevant rabbit anti-human IgG antibody (all at 10 μg/ml) for 45 min at 37°C before opsonization. All strains were washed in PBS and incubated with anti-FH and anti-C3b/iC3b PE conjugated antibodies separately, overnight at 4°C. Yeasts were then washed in PBS and bound FH was detected with an appropriate secondary antibody FITC conjugated for 60 min at RT. All strains were washed in PBS and fixed with PBS/PFA 1%. The yeasts were then fluorescence activated cell sorting (FACS)-gated by SSC vs. FCS density plot and 10,000 events were analyzed. The positive population was detected by one color fluorescence density plot SSC vs. FITC for anti-FH and SSC vs. PE for C3b/iC3b using FACS Calibur and Cell Quest Pro software (BD Biosciences, San Diego, CA, United States).

### Purification of Polymorphonuclear Neutrophils (PMNs)

Fresh blood was obtained from informed consent healthy human donors, based on a vote of the Innsbruck Medical University Ethical Committee. Polymorphonuclear neutrophils (PMNs) were separated from heparinized whole blood by Ficoll Paque-1077 density gradient centrifugation and hypotonic erythrocyte lysis (Monari et al., 2002). The PMNs were collected and resuspended in RPMI medium. The purity of PMNs was determined by light microscopy.

### FACS Analysis of Phagocytosis by PMNs

SN152, *hgt1*Δ/Δ and *hgt1*Δ/Δ*::HGT1* reintegrated strains were grown in YP 0.1% glucose liquid medium ON at 30°C. Yeast cells (1 × 10^6^) were washed in PBS and opsonized with 10% of fresh NHS in PBS for 15 min at 37°C. In a different set up, the parental strain SN152 was blocked with 1 μg anti-Hgt1p or with 1 μg of the chimeric FH6.7/Fc (both at 10 μg/ml) for 45 min at 37°C before the opsonization. Subsequently, all yeasts were washed with PBS and stained with FITC in 0.05 M carbonate-bicarbonate buffer (1 mg/ml) for 15 min at 37°C (Rudkin et al., 2013). Fresh human PMNs and fluorescent *C. albicans* were co-cultured (PMNs : *C. albicans* = 1:10) for 30 min at 37°C/5% CO_2_ in glucose RPMI medium and 0.1%. After co-incubation, cells were centrifuged at 200 *g* to selectively pellet PMNs and remove external yeast cells. The PMNs were fixed in PBS/PFA 1%. Further, the PMNs were gated in SSC vs. FCS density plot and 10,000 events were analyzed. The positive PMNs were detected by one color fluorescence density plot SSC vs. FITC using FACS Calibur and Cell Quest Pro. Phagocytic activity was evaluated as percentage of FITC emission from PMNs, i.e., positive PMNs were those which contained at least on yeast cell.

### PMN Mediated Fungal Killing

Polymorphonuclear neutrophil-mediated killing was assessed by counting colony forming units (CFUs). SN152, *hgt1*Δ/Δ and *hgt1*Δ/Δ*::HGT1* reintegrated strains were grown in YP 0.1% glucose medium overnight at 30°C. The strains were opsonized with fresh 10% NHS/PBS for 15 min at 37°C. In separate experiments, the parental SN152 was blocked with anti-Hgt1p or with FH6.7/Fc (both 1 μg at 10 μg/ml) for 45 min at 37°C and afterward opsonized with 10% NHS/PBS for 15 min at 37°C. Fresh human PMNs and *Candida* were placed in wells of a 96-well plate (PMNs: *C. albicans* = 1:10) in RPMI with 0.1% of glucose. The plate was centrifuged at 800 *g* to promote yeast and PMNs interaction. Subsequently the plate was incubated for 30 min at 37°C, 5% CO_2_ and placed on ice to block further killing. Serial dilutions were carried out by water 0.2% Triton to induce PMNs lysis (Torosantucci et al., 2000). To assess fungal killing, yeasts only were used as CFU control. The percent of killing was evaluated by the following equation (Mota et al., 2015): % of killing = [(CFU control – CFU test)/CFU control] × 100.

### Statistical Analysis

The data reported are expressed as the mean ± standard deviation (SD) from 3 to 5 separate experiments, performed at different days. The data were analyzed by unpaired *t*-test, or, when comparing more than two groups, with one-way ANOVA using the parental strain as reference which allows sample-to-sample *p*-values. In all experiments a *p*-value of < 0.05 was considered statistically significant.

### Ethics Statement

The Ethics Committee of the Medical University of Innsbruck confirmed that ethics committee approval is not required for the use of anonymised leftover specimens from blood donations of the local blood bank, for scientific purposes.

## Results

### Glucose Dependent *HGT1* Expression

**FIGURE 2:**
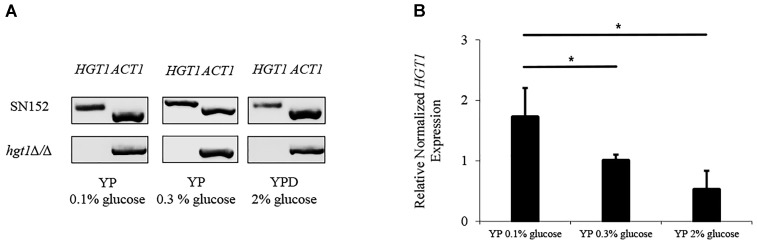
Glucose dependent *HGT1* expression. **(A)** Electrophoresis of qPCR products (cropped figure from same experiment, Supplementary Figure S1). **(B)** Expression of *HGT1* normalized to *ACT1*. The electrophoresis run shown is a representative of three separate experiments. *HGT1* expression was normalized to *ACT1*, a constitutively expressed housekeeping gene that did not show significant differences in expression at different glucose concentrations. The graph shows the average of three separate experiments. Bars represent the mean + SD. Significance (^∗^*p* < 0.05) was determined by one-way ANOVA, using SN152 for comparison.

A quantitative real time PCR was performed to analyze regulation of *HGT1* by glucose. The mRNA of parental SN152 and *hgt1*Δ/Δ null mutant strains were extracted and amplified by specific primers for *HGT1* and *ACT1*. In order to confirm an absence of signal from the *hgt1*Δ/Δ mutant, the qPCR amplification was visualized by electrophoresis (Figure 2A and Supplementary Figure S1). The expression of *HGT1* was evaluated by Bio-Rad CFX manager software as a relative normalized expression by using *ACT1* as a housekeeping gene. *HGT1* gene expression in low glucose medium (0.1%) was significantly higher compared to high glucose medium (0.3 and 2%) (Figure 2B).

### Distribution of Hgt1p on *C. albicans* and in Vesicle Trafficking: Hgt1p Present in Released Exosomes

**FIGURE 3:**
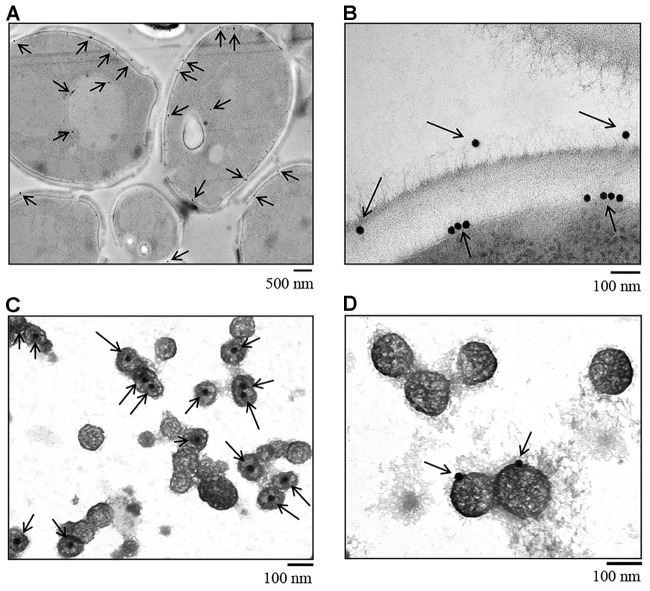
Immunogold staining of *C. albicans* SN152 grown in physiological low glucose medium (0.1%) after HPF cryofixation visualized by TEM. **(A)** Intracellular distribution and Hgt1p detection in the cell membrane and cell wall of *C. albicans.* **(B)** Zoomed-in view of Hgt1p in the cell membrane and on the cell wall surface. **(C)** Gold staining of secretory pathway vesicles showing Hgt1p protein in the internal part of the vesicle and **(D)** on exosomes released by *C. albicans* to the environment, demonstrating the presence of Hgt1p integrated in the vesicle membrane.

*Candida albicans* SN152 strain cryofixed by HPF was stained with colloidal gold-labeled anti-Hgt1p antibody and subsequently visualized by TEM to assess Hgt1p distribution on yeast. Hgt1p (visualized by black dots) was detected intracellularly, in the cell membrane and on the cell wall of *C. albicans* (Figure 3A), especially when focussing on cell membrane and cell wall (Figure 3B). Inside the fungal cell, Hgt1p was clearly associated with purified secretory vesicles, in the internal part of the vesicles (Figure 3C). In contrast, in exosomes released by *C. albicans* to the environment, Hgt1p was detected within the vesicular membrane (Figure 3D). About 50% of the intracellular vesicles were positive for Hgt1p, in contrast to only 30% in released exosomes. Specificity of antibody staining was determined using the *hgt1*Δ/Δ null mutant that was stained with anti-Hgt1p and gold-labeled secondary antibody and with the SN152 parental strain staining using only the secondary gold-conjugated antibody. The missing black dots in *hgt1*Δ/Δ or the incubation of only the secondary antibody with the parental strain confirm the specificity of anti-Hgt1p and exclude cross-reactions (Supplementary Figure S2).

### Deposition of FH and C3b/iC3b on *C. albicans* Surface

**FIGURE 4:**
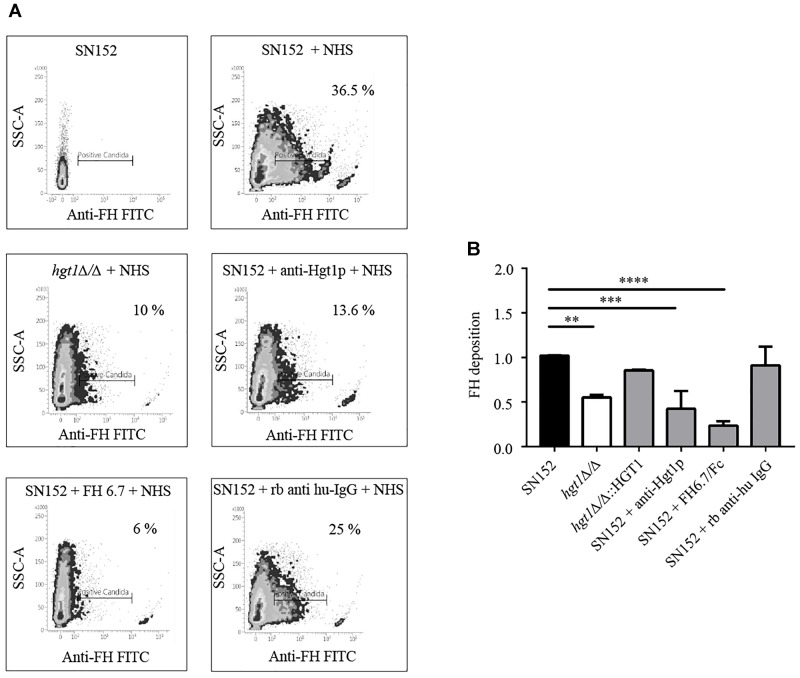
Factor H (FH) binding to *C. albicans*. **(A)** Binding of FH present in NHS to *C. albicans* was analyzed by FACS. Density plots from one representative experiment are shown. X-axis, fluorescence of FH binding. Gates represent the % of *C. albicans* cells that stain positively for FH. Y-axis, side scatter (SSC). The components of the reaction mixture are indicated above each graph. **(B)** Cumulative data of FH binding to *C. albicans*. FH binding to SN152, *hgt1*Δ/Δ, *hgt1p*Δ/Δ*::HGT1* reintegrated strain, or SN152 pre-incubated with anti-Hgt1p or the chimeric molecule FH6.7/Fc, or with irrelevant rabbit anti-human IgG. Data are expressed as mean fluorescent intensity (MFI) normalized to SN152 parental strain. Bars represent the normalized mean fold-increase + SD of five separate experiments ^∗∗^*p* < 0.01, ^∗∗∗^*p* < 0.001, ^∗∗∗∗^*p* < 0.0001, by one-way ANOVA, using SN152 for comparison.

**FIGURE 5:**
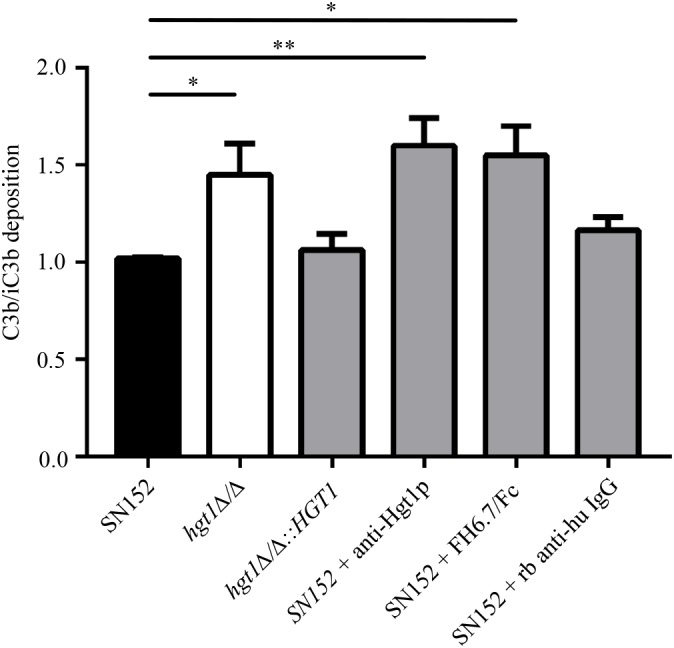
Decreased FH binding is associated with increased C3b/iC3b deposition on *C. albicans.* Relative C3b/iC3b deposition on SN152 parental (black bar), *hgt1*Δ/Δ (white bar), *hgt1*Δ/Δ*::HGT1* (complemented strain), each incubated with NHS, or on SN152 incubated with anti-Hgt1p or with the chimeric molecule FH6.7/Fc to block FH binding, or with irrelevant rabbit anti-human IgG. Data are expressed as MFI normalized to SN152 parental strain. Each bar represent the normalized mean + SD of five separate experiments ^∗^*p* < 0.05, ^∗∗^*p* < 0.01, by one-way ANOVA, using SN152 for comparison.

As revealed by FACS analysis, a significantly higher proportion of parental SN152 yeast cells bound FH compared to the *hgt1*Δ/Δ null mutant or SN152 pre-incubated with anti-Hgt1p or by the chimeric molecule FH6.7/Fc (Figure 4A). The *hgt1*Δ/Δ null mutant strain bound significantly (approximately 40%) less FH than the parental strain SN152. Preincubation of the latter with anti-Hgt1p or the chimeric molecule FH6.7/Fc also significantly lowered FH binding by ∼60 or ∼80%, respectively (Figure 4B). Accordingly, C3b/iC3b deposition on the surface of the *hgt1*Δ/Δ null mutant strain was significantly higher compared to the parental strain (approximately 1.5 times). Blocking FH binding to SN152 using anti-Hgt1p or FH6.7/Fc also significantly increased (by a factor of ∼1.5) C3b/iC3b deposition on the yeast surface compared to the parental strain. SN152 treated with rabbit anti-human IgG of irrelevant specificity showed similar C3b/iC3b deposition as the parental strain (Figure 5).

### *In vitro* Phagocytosis by PMNs

**FIGURE 6:**
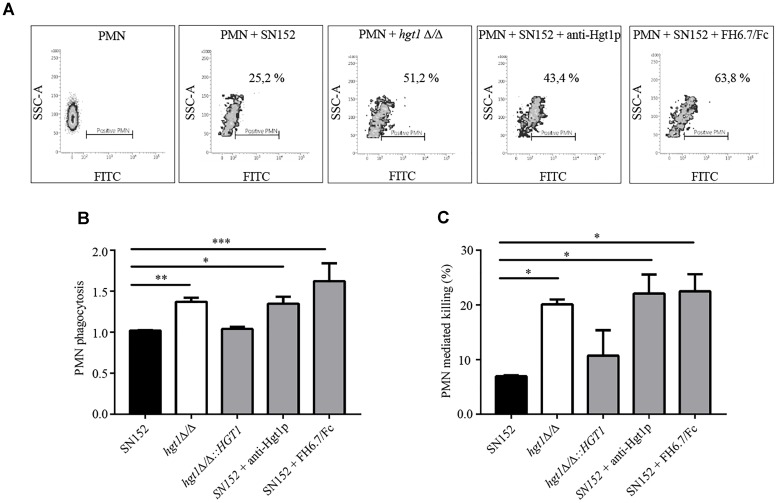
Factor H binding contributes to evasion of phagocytosis by PMNs of *C. albicans* at physiological glucose concentrations. **(A)** Scatter (density) plots from a representative experiment showing association of FITC-labeled yeast with PMNs as measured by FACS. The density plots show fluorescence (FITC) on the X-axis as a measure of PMNs with associated *C. albicans*. The Y-axis shows side scatter. The constituents of each reaction are indicated above each histogram. All reactions contain NHS. **(B)** Cumulative data to show that loss or decreased FH binding to *C. albicans* is associated with increased phagocytosis. **(C)** PMN medicated killing performed under similar conditions as indicated in **(B)**. Bars represent the normalized mean + SD of four separate experiments. The % of PMNs with associated yeast in **(B)** is normalized to PMNs co-incubated parental strain SN152 plus NHS. ^∗^*p* < 0.05, ^∗∗^*p* < 0.01, and ^∗∗∗^*p* < 0.001, by one-way ANOVA, using SN152 for comparison.

Fluorescence activated cell sorting analysis showed that a significantly lower fraction of freshly isolated human PMNs (25.2%) were associated with NHS-opsonized SN152 (parental strain) compared to NHS-opsonized *hgt1*Δ/Δ null mutant (51.2%) after 30 min of incubation (Figure 6A; compare graphs labeled PMNs + SN152 and PMNs + *hgt1*Δ/Δ). Incubation of non-opsonized fungi with PMNs resulted in minimal association with PMNs (below 5%, data not shown), demonstrating the need for complement for opsonophagocytosis of *Candida*. Blocking FH binding to the parental strain SN152 with anti-Hgt1p or the chimeric molecule FH6.7/Fc both increased association of fungi with PMNs [Figure 6A; compare PMNs + SN152 + anti-Hgt1p (43.4% PMNs positive) and PMNs + SN152 + FH6.7/Fc (63.8% of PMNs positive) with PMNs + SN152 (25.2% PMNs positive)]. The cumulative data from five separate experiments [as the % of PMNs associated with fungi relative to the mixture that contained NHS-opsonized SN152 incubated with PMNs (black bar)] is shown in Figure 6B.

Confocal microscopy was carried out to confirm that the association of *C. albicans* with PMNs in the FACS assay in Figure 6 translated to phagocytosis. The *hgt1*Δ/Δ null mutant showed a higher extent of fluorescent *C. albicans* cells internalized in comparison to internalization of parental strain SN152; the number of PMNs that internalized 1, 4, or 5 *hgt1*Δ/Δ null mutant cells compared to the parental strain reached statistical significance (Supplementary Figure S3).

To confirm that the FITC fluorescence emission from PMNs represents the phagocytosis activity (i.e., internalization), an additional staining by Uvitex 2B was carried out in a separate set up of experiments to discriminate the internalized (green) from the PMN-attached yeast cells (blue). The tubes were placed on ice and the samples were stained with 1 μl of Uvitex 2B (0.01% in PBS) for 1 min. FACS analysis confirmed that 16% of PMNs had phagocytosed yeast cells and 5% only carried yeast cells attached to the membrane (Supplementary Figure S4).

### Killing Assay

*Candida albicans* killing by human PMNs was significantly more pronounced for *hgt1*Δ/Δ and SN152 blocked by anti-Hgt1p or FH6.7/Fc (each approximately 20%) when compared to the untreated parental strain (Figure 6C).

## Discussion

In the present study we increased the knowledge on Hgt1p expression showing a higher expression of *HGT1* in media with low glucose concentration (0.1%) – not unexpected for a glucose transporter – which resembles the physiological concentration in humans (Buu and Chen, 2014). This concentration was therefore used for all following experiments. In this respect, it is important to mention that several *in vitro* mycology studies use YPD, which contains a completely unphysiological glucose concentration (of 2%), even for diabetic humans.

Hgt1p was detected within the cell (Figures 3A–C), the cell membrane and cell wall (Figures 3A,B) and was associated with both intracellular and extracellular vesicles (Figures 3C,D). Based on these findings it is likely that Hgt1p is being transported in intracellular secretory vesicles and accumulates not only in the cell membrane, but also on the cell wall, which is corroborated by the fact that 60 nm AmBisome liposomes can transit intact through the *C. albicans* cell wall (Walker et al., 2018). One may hypothesize that Hgt1p plays not only a canonical transmembrane role in glucose transport, but, when it binds FH on the surface of the yeast cell wall, it serves an additional non-canonical role as a moonlighting protein (Karkowska-Kuleta and Kozik, 2014), by interfering with host cells, enabling *C. albicans* to evade the immune attack, detailed below.

During co-evolution *C. albicans* has developed virulence factors, such as FH binding molecules, enabling it to functionally mimic human cells (Phan et al., 2007), thereby limiting the attack of the immune system, e.g., complement, during blood dissemination (Luo et al., 2011). Examples are *C. albicans* binding to plasminogen, increasing its ability to disseminate (Crowe et al., 2003), or binding to C4BP, enabling it to regulate complement classical and lectin pathways on its surface (Meri et al., 2004).

Factor H represents the main inhibitor of the alternative pathway of complement and *C. albicans* has evolved FH binding molecules on its surface (Poltermann et al., 2007; Luo et al., 2010, 2013). At least one of these FH binding molecules, pH-regulated antigen 1 (Pra1), can be released into the host environment to inhibit the immune response by binding to C3 and preventing its conversion (Luo et al., 2010). In a previous study, the transmembrane protein Hgt1p, was identified as a FH binding molecule in *C. albicans* by screening a cDNA expression library (Lesiak-Markowicz et al., 2011). Hgt1p is characterized by 12 transmembrane domains and plays its canonical role in glucose metabolism on the cellular membrane (Fan et al., 2002). A null mutant strain, *hgt1*Δ/Δ, has been constructed earlier and, similar to our current observations, also showed a decrease in FH binding (Lesiak-Markowicz et al., 2011). These data together confirm the non-canonical role of the Hgt1p as FH binding molecule (Lesiak-Markowicz et al., 2011), and establish its role as a virulence factor by inhibiting the complement system on *C. albicans*.

In order to assess whether the blocking of Hgt1p is a strategy to limit the complement inhibition, an antibody able to detect an epitope in the extracellular part of Hgt1p was generated commercially. To study the effects of the complement inhibition by Hgt1p, we focussed on FH deposition, C3b/iC3b conversion and deposition on the yeast’s surface, phagocytosis, and killing by human PMNs. Blocking FH-Hgt1p interactions by a specific antibody, resulted in FH displacement (Figure 4B) and an increased C3b/iC3b conversion and deposition on the surface (Figure 5), similar to that displayed by *hgt1*Δ/Δ. FH domains 6 and 7 encompass the sites of binding used by streptococci (Blackmore et al., 1998), *Neisseria* (Madico et al., 2006) and non-typeable *Haemophilus influenzae* (Wong et al., 2016) among other pathogens to inhibit the complement alternative pathways (Blom et al., 2017). We therefore used a chimeric molecule, consisting of human FH domains 6 and 7 fused with the human IgG Fc receptor (Shaughnessy et al., 2014), and demonstrated that this functions as another useful FH blocker thereby confirming our initial hypothesis. However, incubations with blockers did not affect growth (Supplementary Figure S5).

FH6.7/Fc has shown efficacy against *N. meningitidis* (Shaughnessy et al., 2014), *N. gonorrhoeae* (Shaughnessy et al., 2018), non-typeable *Haemophilus influenzae* (Wong et al., 2016), and group A streptococci in rodent models (Blom et al., 2017). Our data support further evaluation of FH6.7/Fc as an immunotherapeutic against *C. albicans*. Activation of the classical pathway through the Fc domains present in anti-Hgtp1 and FH6.7/Fc may contribute to C3b/iC3b deposition and phagocytosis through FcγR engagement, in addition to enhancement of alternative pathway activation by blocking FH access to the fungal surface. Compared to anti-Hgt1p, FH6.7/Fc was slightly more effective in preventing FH deposition and enhancing phagocytosis which may be due to the fact that this chimeric molecule may also target other FH binding molecules expressed by *C. albicans* (Poltermann et al., 2007; Luo et al., 2010, 2013). However, the presence of Hgt1p not only on the cell membrane and cell wall, but also on secreted exosomes may interfere with this evasion mechanism, acting as a decoy that diverts FH away from fungal surface.

Phagocytosis by human PMNs was investigated as it represents the first line of innate immune defense against pathogens, such as *C. albicans* (Gazendam et al., 2016). The αMβ2 integrin (β2 INT) CD11b/CD18 or complement receptor 3 (CR3) on PMNs plays a major role in increasing the adhesion to *C. albicans* by β-glucan binding (O’Brien and Reichner, 2016). It also binds to the iC3b which opsonises the yeast during complement attack (Ueda et al., 1994). The recognition of the fungal cell wall constituents together with the deposited iC3b generates a marked response toward the yeast, mediated by transduction pathways and leading to PMN-mediated fungal killing (Gazendam et al., 2016).

Our previous study revealed that Hgt1p is a CR3 analog (Lesiak-Markowicz et al., 2011). The specific iC3b binding to a CR3 like molecule on *C. albicans* may limit iC3b surface deposition and thus also phagocytosis. In order to assess whether FH blocking represents a tool to increase the fungal killing, a co-culture of all yeast strains used in this study with fresh PMNs from healthy human donors was undertaken. The results revealed a significant higher degree of *hgt1*Δ/Δ phagocytosis in comparison to that of the parental strain. When specific blockers were applied to the parental strain, *in vitro* phagocytosis was similar to that induced by the *hgt1*Δ/Δ null mutant. This may imply that appropriate antibodies might be able to limit FH access and increase C3b/iC3b deposition and phagocytosis of the yeast.

In order to assess whether the increased phagocytosis of the parental strain coated by anti-Hgt1 is due to the blockage of Hgt1p-FH binding or due to the Fc receptor recognition by PMNs, single Fab fragments of the antibody were generated which also significantly increased phagocytosis of the parental strain to a similar extent as anti-Hgt1p whole antibody (data not shown).

Even more pronounced phagocytosis by PMNs was observed using the chimeric molecule FH6.7/Fc, confirming the role of FH binding molecules on *C. albicans* as complement evasion molecules. The killing assay confirmed the phagocytosis results and corroborated the role of Hgt1p as virulence factor. High expression of Hgt1p was also detected by immunofluorescence on SN152 hyphae (data not shown). Blocking of Hgt1p may thus also represent an additional tool to prevent tissue invasion by *C. albicans.* All these findings support further testing in a mouse infection model.

So far there are no antibody-based therapies available in the treatment of *C. albicans*. Mycograb (NeuTec Pharma, a subsidiary of Novartis AG, Basel, Switzerland) is a human-derived single chain recombinant antibody fragment against *C. albicans* heat shock protein 90 (Hsp90), a stress induced protein (Burt et al., 2003) involved in intracellular processes, but also detected on the cell wall (Chaffin et al., 1998). Several *in vitro* studies have shown that the effect of classic antifungal treatment can be enhanced by Mycograb, which decreases the minimal inhibiting concentration (MIC) of the yeast to antifungals (Bugli et al., 2013). This combination between antifungals and Mycograb has decreased mortality in patients with invasive *Candida* infection in a small randomized trial (Pachl et al., 2006), but it is still not approved for clinical use.

**FIGURE 7:**
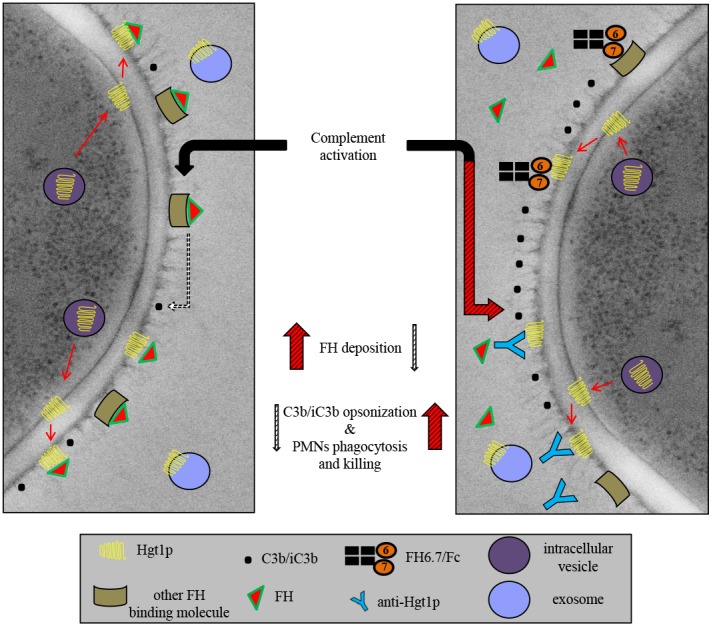
Schematic representation of Hgt1p complement inhibition model. Hgt1p is carried to the cell wall by exosomes. The protein crosses the cell wall and acts as a FH binding molecule. Blockage of FH binding and the resulting up-regulation of C3b/iC3b conversion and deposition on fungal surface increases phagocytosis by PMNs.

The blocking of evasion on the fungal surface (Figure 7) may represent a first step toward developing more specific ways to treat human infection caused by fungi. Hgt1p may form a starting point for novel antifungal therapies that target specific molecules involved in pathogenesis.

## Author Contributions

SK designed and performed the experiments, analyzed the data, and wrote the manuscript. CS, GR, UB, SC, RC, HH, CL-F, and DO-H analyzed the data. JS, SR, NG, and RW analyzed the data and wrote the manuscript.

## Conflict of Interest Statement

The authors declare that the research was conducted in the absence of any commercial or financial relationships that could be construed as a potential conflict of interest.
