# Supplementary figures and images for: Candida albicans Factor H Binding Molecule Hgt1p – A Low Glucose-Induced Transmembrane Protein Is Trafficked to the Cell Wall and Impairs Phagocytosis and Killing by Human Neutrophils
Source: Front Microbiol. 2019 Jan 15;9:3319. doi: 10.3389/fmicb.2018.03319 (PMC6340940; doi:10.3389/fmicb.2018.03319)

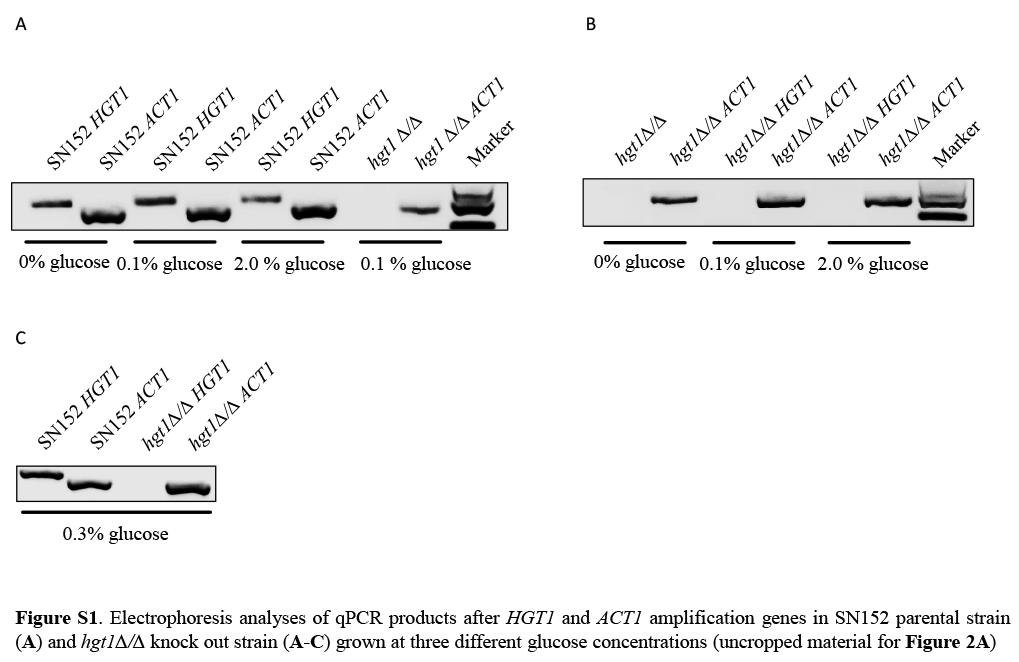

Supplement: Supplementary file 1 [file Image_1.tif]

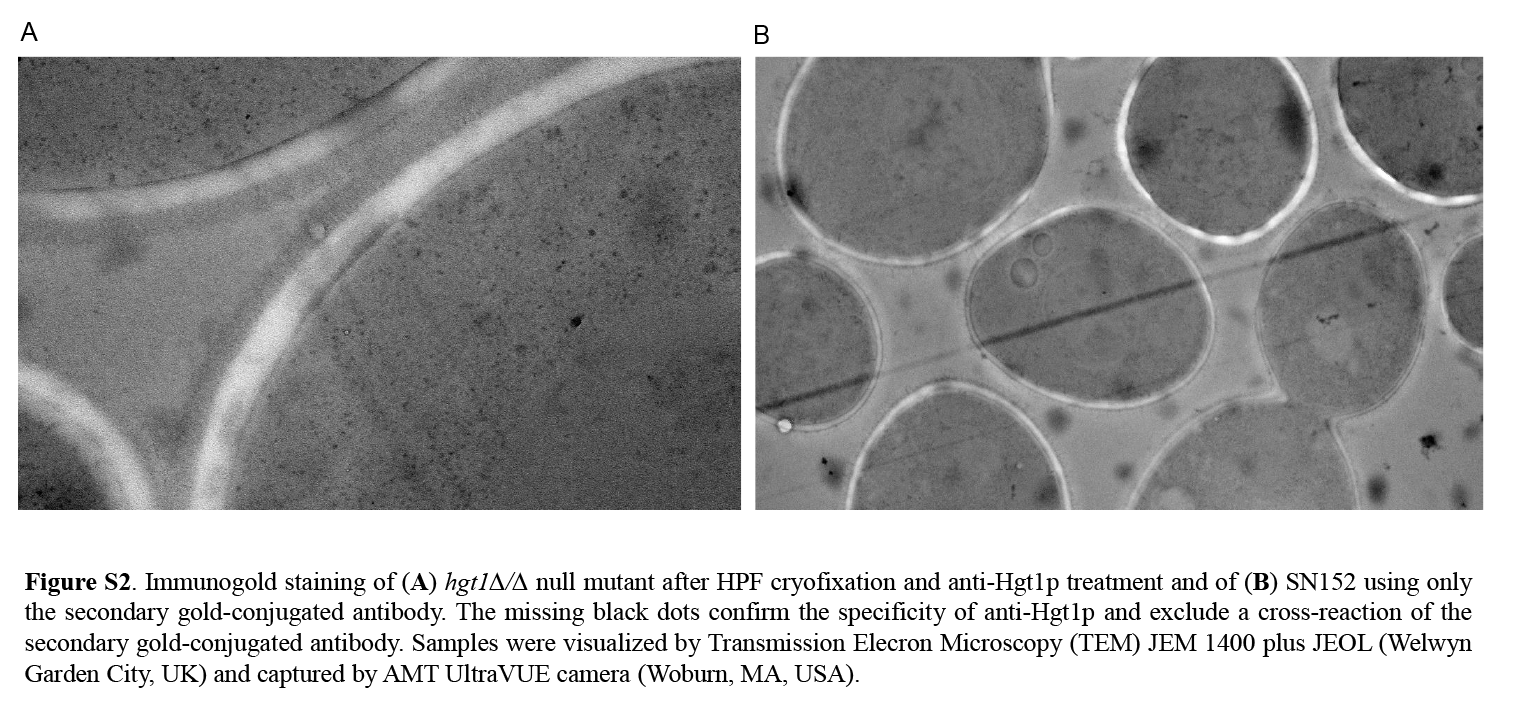

Supplement: Supplementary file 2 [file Image_2.TIF]

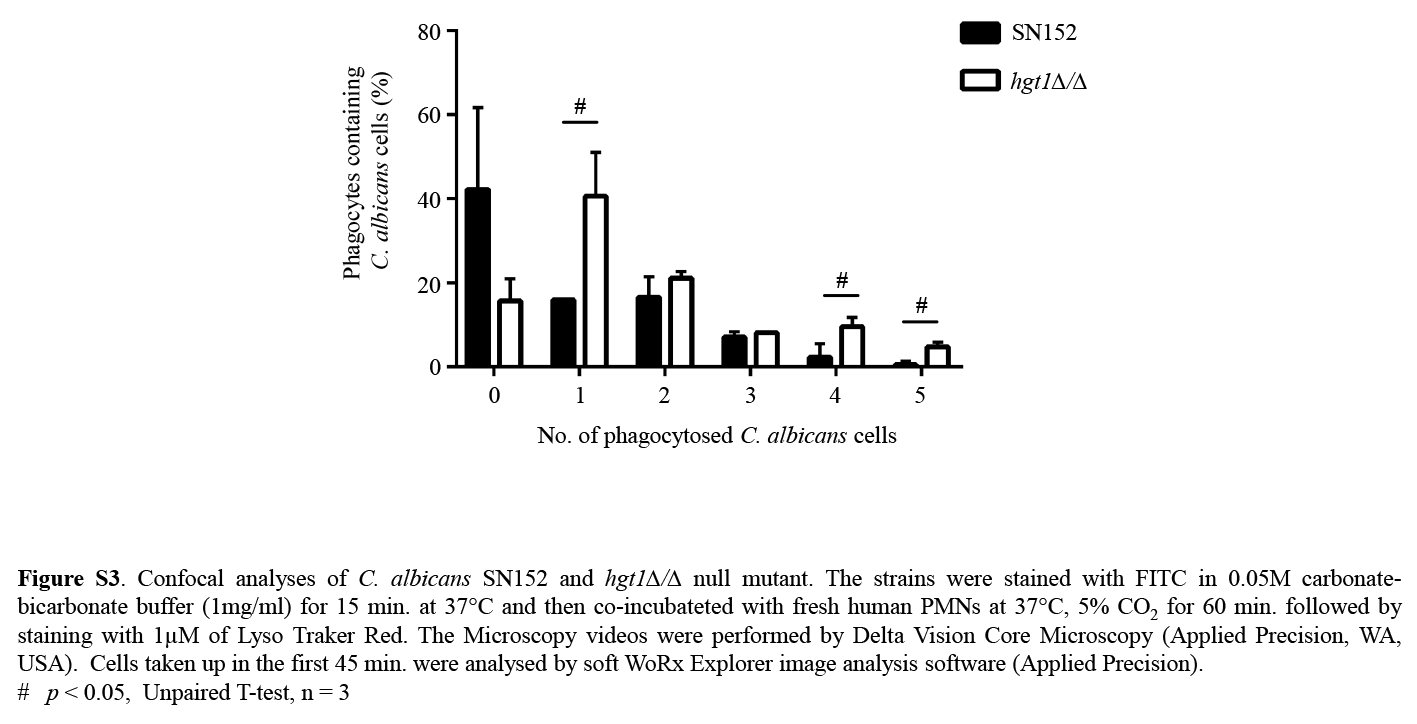

Supplement: Supplementary file 3 [file Image_3.TIF]

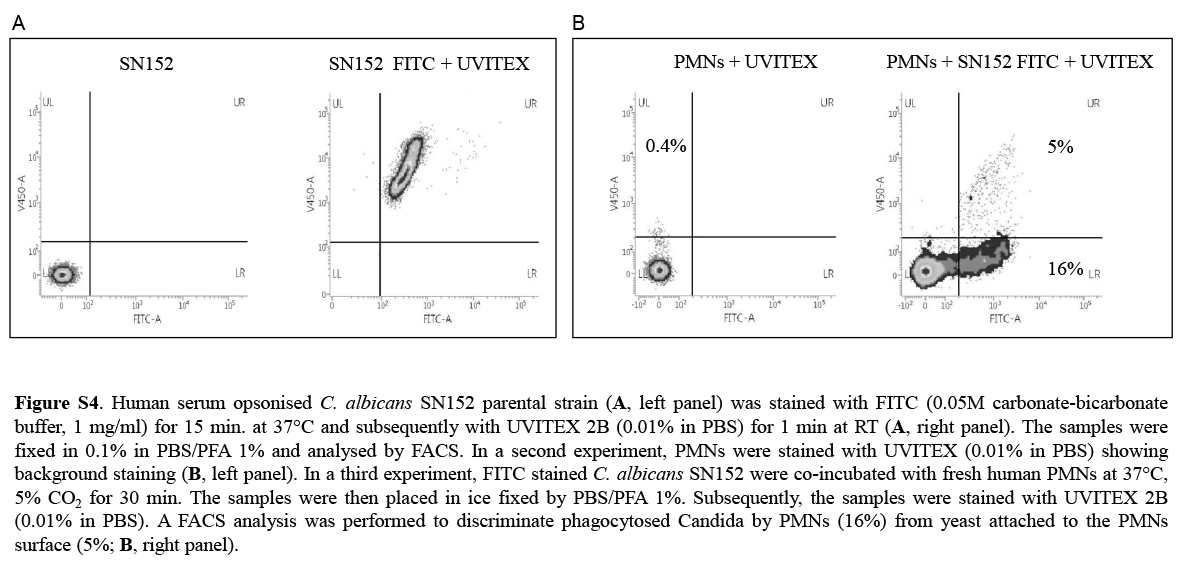

Supplement: Supplementary file 4 [file Image_4.tif]

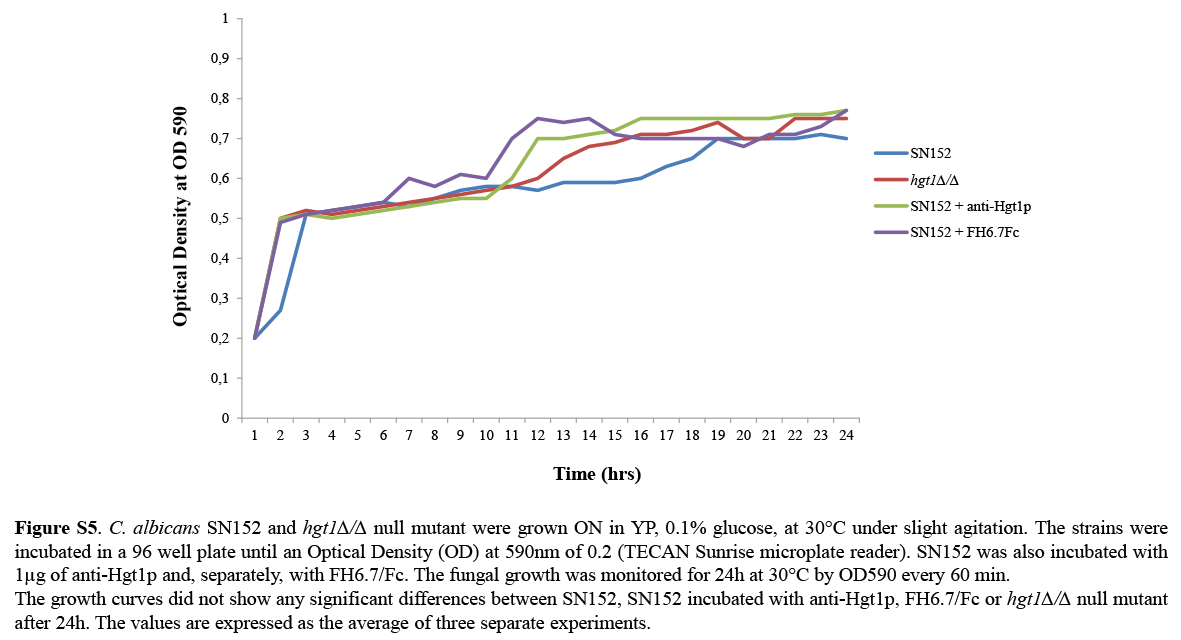

Supplement: Supplementary file 5 [file Image_5.tif]
